# A Green and Sustainable Approach for Melatonin Determination: Comparative Assessment of Hybrid Micellar Liquid Chromatography and Differential Pulse Voltammetry

**DOI:** 10.1021/acsomega.6c01692

**Published:** 2026-05-21

**Authors:** Zehra Üstün, Ümran Sofu, İlkay Konçe, Ebru Çubuk Demiralay

**Affiliations:** † 52994 Suleyman Demirel University, Atayalvaç Vocational School of Health Services, Medical Services and Techniques Department, 32400 Isparta, Türkiye; ‡ Suleyman Demirel University, Faculty of Pharmacy, Department of Analytical Chemistry, 32000 Isparta, Türkiye

## Abstract

Melatonin, the main indolamine compound in the pineal gland, which plays a role in the regulation of biological rhythms and shows promise in the treatment of sleep disorders, is widely used today. In this study, the determination of melatonin in tablet formulations was comparatively evaluated using hybrid micellar liquid chromatography (HMLC) and differential pulse voltammetry (DPV) methods. The compound was analyzed by the HMLC method in a water–ethanol binary mixture containing 0.05 M sodium dodecyl sulfate and 20% ethanol (v/v) adjusted to pH 7.5. It was then determined by the DPV method using a gold electrode in Britton-Robinson buffer (pH 5.0). The results were systematically analyzed, the analytical methods were validated, and a comprehensive comparison was conducted. Under optimized conditions, the HMLC method yielded melatonin concentrations between 2.0 and 8.0 μg/mL (*r* = 0.999); the DPV method showed a linear calibration range between 1.9 and 139.4 μg/mL (*r* = 0.997). The LOD values for the HMLC and DPV methods were 0.335 μg/mL and 0.481 μg/mL, respectively; the LOQ values were 1.016 μg/mL and 1.456 μg/mL, respectively. The mean recovery was 99.57% for HMLC and 100.39% for DPV. The suitability of the developed methods for the 12 principles of green analytical chemistry (GAC) and the white analytical chemistry (WAC) approach was evaluated using established tools. This study presents, for the first time, a comparative evaluation of HMLC and DPV methods for melatonin determination within the framework of green and white analytical chemistry, using a nontoxic micelle mobile phase and an unmodified gold electrode.

## Graphical abstract

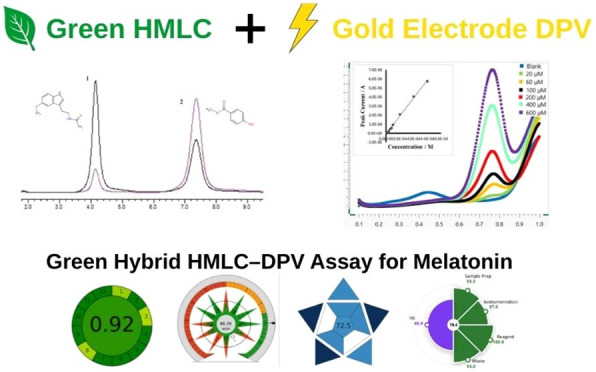

## 1 Introduction

Melatonin (*N*-acetyl-5-methoxytryptamine) is an endogenous hormone synthesized primarily by the pineal gland in response to the circadian rhythm. It is crucial in regulating sleep-wake cycles, immune responses, and antioxidant defense mechanisms.^1,2^ Its strong antioxidant properties reduce oxidative damage by scavenging reactive oxygen species (ROS).^3^ Given its widespread therapeutic applications, melatonin is frequently used as an over-the-counter supplement for sleep disorders, jet lag, and neurodegenerative diseases.^4^ The clinical importance and widespread consumption of melatonin necessitate the development of robust, accurate, and sustainable analytical methods to ensure the quality and safety of pharmaceutical products.

High-performance liquid chromatography (HPLC) and voltammetric methods are used in melatonin determination due to their high sensitivity, accuracy, cost-effectiveness, and fast response times.^5–8^ In analyses performed with reverse-phase liquid chromatography (RPLC), a commonly used HPLC mode, tetrahydrofuran (THF), acetonitrile (ACN), and methanol (MeOH) are frequently used due to their high elution strengths. The use of large quantities of these organic solvents in RPLC analyses of compounds with low water solubility generates significant waste. To minimize waste toxicity, environmentally friendly solvents should be preferred. Due to environmental concerns, the selection of a solvent compatible with green and white analytical chemistry principles is important for chromatographic performance.^9,10^ The use of ethanol (EtOH), a biodegradable and renewable solvent, offers an environmentally friendly alternative to conventional solvents.^10,11^ Since EtOH has a lower vapor pressure than ACN or MeOH, respiratory exposure is lower, and it is safer for analysts. However, its high viscosity will increase back pressure in HPLC systems, method optimization is necessary.^12^ Interest in green chemistry is growing with the micellar liquid chromatography (MLC) method, used as an alternative to the classical RPLC method.^13–15^ In this method, surfactants (sodium dodecyl sulfate, SDS, cetyltrimethylammonium bromide, etc.) are used above the critical micelle concentration (CMC). HMLC, which integrates MLC and conventional RPLC, reduces the use of organic solvents in analyses. This approach typically uses a surfactant in the mobile phase and a more environmentally friendly organic solvent. The main advantage of this approach is the formation of micelles and premicelle aggregates that alter chromatographic retention. This increases selectivity in the separation of analytes.^16–19^ In RPLC analyses performed using green and white analytical methods, HMLC, a more environmentally friendly method, was chosen. In the study conducted by Daldal and Çubuk Demiralay, EtOH-water binary mixtures containing 11%, 13%, 15%, and 20% (v/v) EtOH were used to compare the analytical performance of ranitidine and famotidine with green RPLC and HMLC methods. Although the liquid chromatographic behavior was similar for famotidine and ranitidine in both methods, lower retention time (*t*_*R*_) values were obtained with the HMLC method. This situation made the HMLC method, which was improved by adding SDS as a surfactant to EtOH-water binary mixtures, even more environmentally friendly.^19^

Electroanalytical methods are widely preferred for the selective and sensitive determination of target compounds, as they often eliminate the need for extensive sample pretreatment typically required in chromatographic and spectroscopic techniques.^20^ Among these, DPV stands out due to its simplicity, low cost, and short analysis time.^21^ It enables the detection of analytes at trace levels with high sensitivity and selectivity, making it particularly suitable for monitoring electroactive species in complex media.^22^ Moreover, DPV is especially advantageous for irreversible systems, where slow electron transfer kinetics at the electrode surface benefit from the technique’s enhanced discrimination against background currents, resulting in improved detection limits.^23^ Furthermore, since the analyses are mostly performed in aqueous environments, electrochemical techniques become an environmentally friendly analytical alternative.^24,25^ Among the various working electrodes employed in electrochemical methods, gold electrodes are widely used in electroanalytical studies owing to their exceptional stability, broad potential ranges, and fast electron-transfer kinetics.

This study presents comprehensive data integrating the 12 principles of GAC and WAC approach for the qualitative and quantitative determination of melatonin. Using the capacity factor (*k*) values of melatonin at the investigated mobile phase pH values for the optimization of the HMLC method, the hydrophobicity descriptor (φ_0_) will be calculated for the first time to provide information about the hydrophobicity of the compound.^26^ The aim is to validate the optimized HMLC and DPV methods in accordance with the International Council for Harmonisation of Technical Requirements for the Registration of Pharmaceuticals for Human Use (ICH Q2­(R2)) guidelines and to perform quantitative analysis of pharmaceutical tablet formulations.^27^ Additionally, the study aimed to demonstrate the practical applicability of the developed methods and their potential for routine quality-control analyses of melatonin in pharmaceutical formulations.

## 2 Materials and Methods

### 2.1 Chemicals and Reagents

All chemicals were of analytical reagent grade and were used as received without additional purification. Melatonin and its pharmaceutical dosage form, Ocean Melatonin (3 mg per tablet), were kindly supplied by Orzaks İlaç ve Kimya San. Tic. A.Ş. (Türkiye). The internal standard (IS) methylparaben, uracil, and all other chemicals used in the preparation of the solutions in this study were purchased from Sigma-Aldrich (St. Louis, USA).

### 2.2 Instrumentation

All chromatographic analyses were performed using a Shimadzu i-Series HPLC system (Model LC-2050C 3D, Kyoto, Japan) equipped with a quaternary solvent delivery unit, an autosampler, a column oven with forced-air circulation, and a photodiode array (PDA) detector. The system was controlled by LabSolutions software and operated under fully automated conditions. A Mettler Toledo (Zurich, Switzerland) pH meter and a combined Ag/AgCl glass electrode (Mettler Toledo Inlab 413) were used for pH measurements of the prepared mobile phases. In all electrochemical measurements, a conventional three-electrode cell was used, with an unmodified gold electrode (BASi, 3 mm diameter) as the working electrode, an Ag/AgCl electrode (BASi, 3.0 M NaCl) as the reference electrode, and a platinum wire (BASi) as the counter electrode. Voltammetric measurements were performed using an Autolab IMP potentiostat/galvanostat (Metrohm), and the experimental data were processed and analyzed using NOVA 2.1.8 software.

### 2.3 Chromatographic Conditions

For optimum mobile phase and robustness tests, EtOH-water binary mixtures containing 15%, 20%, and 25% (v/v) EtOH were prepared. The pH values of the mobile phases containing 40 mM o-phosphoric acid were adjusted to 7.0, 7.5, and 8.0 with a 1 M NaOH solution. o-phosphoric acid was used as a buffer component because of its appropriate dissociation constant (p*K*_a_) values and the symmetrical peak shape of these compounds in this buffer solution. SDS was used in the prepared mobile phases in the range of 0.03 to 0.07 M. Analyses with an injection volume of 20 μL were performed using a Supelco Ascentis RP-Amide column (150 × 4.6 mm, 5 μm). All analysis were carried out at a constant temperature of 37 °C and a flow rate of 1 mL/min. Melatonin and methylparaben (IS) were analyzed at 220 and 254 nm, respectively.

The correct pH value for EtOH-water binary mixtures was determined according to IUPAC guidelines^28^ using the specified reference pH of the buffer solution used.^29^ pH standardization in these hydroorganic mixtures was carried out using 0.05 mol/kg primary standard reference solution (KHP).^28–30^

### 2.4 Preparation of Gold Electrode

Prior to each measurement, the electrode surface was carefully prepared to ensure reproducible results. The gold electrode was rinsed with distilled water and polished on a microcloth with an alumina slurry, followed by additional polishing with a few drops of distilled water to achieve a uniform surface. After thorough rinsing with double-distilled water, the electrode was subjected to electrochemical pretreatment by cycling the potential between 0.0 and +1.0 V at a scan rate of 100 mV s^–1^ for 3 cycles in the supporting electrolyte, aiming to reduce background currents and widen the effective potential window.

### 2.5 Preparation of Stock, Standard, and Sample Solutions

For voltametric experiments, a 232.28 μg/mL melatonin stock solution was prepared and stored in the dark at refrigerated temperature until use. The required concentrations were obtained by appropriate dilution with the supporting electrolyte prior to measurement. For chromatographic experiments, standard solutions of melatonin and methylparaben were prepared by dissolving them in the mobile phase at a concentration of 50 μg/mL and stored at 4 °C. Working solutions were prepared by appropriate dilution of the stock solutions. The compounds *k* values were determined using a 50 μg/mL uracil solution. The IS was maintained at a constant concentration of 2 μg/mL throughout the study.

Britton-Robinson buffer (BRB, 0.04 M) solutions with a pH range of 4.0–9.0 were prepared by mixing CH_3_COOH, H_3_BO_3_, and H_3_PO_4_ in equal concentrations. Phosphate buffer (PB, 0.1 M) solutions were prepared using appropriate mixtures of Na_2_HPO_4_ and NaH_2_PO_4_ for the pH range of 6.0–8.0. Acetate buffer (AB, 0.1 M) solutions at pH 3.5, 4.5, and 5.5 were prepared using CH_3_COOH. The pH of all buffer solutions was adjusted with 5 M NaOH as required.

### 2.6 Preparation of Tablet Solution

To determine the active ingredient of melatonin in tablet formulation, 10 tablets were crushed in a porcelain mortar. For voltammetric analysis, tablet stock solutions were prepared by weighing an amount of tablet powder equivalent to a 232.28 μg/mL melatonin concentration and transferring it into a volumetric flask, after which the volume was made up to 10 mL with double-distilled water. The mixture was sonicated for 10 min and then allowed to stand at room temperature for 15 min to enable the sedimentation of insoluble excipients. Working solutions were prepared by taking appropriate aliquots of the supernatant and diluting them with the selected supporting electrolyte prior to measurement. No filtration step was applied for voltammetric measurements of tablet solutions, and the supernatants were used directly.

For chromatographic analysis, tablet powder equivalent to 1 tablet was weighed and taken into a 100 mL volumetric flask. A portion of the mobile phase was added, and the mixture was sonicated; the volume was then adjusted with the mobile phase. This solution was filtered through a blue band filter paper and diluted at different concentrations so that melatonin was within the determined linear calibration range.

### 2.7 System Suitability Test

System suitability tests (SST) were performed to demonstrate the overall performance of the liquid chromatographic system and to demonstrate that the method can provide accurate and reproducible results under defined experimental conditions. SST was performed at a concentration of 5 μg/mL (which lies within the calibration range) by injecting the standard solution under the determined chromatographic conditions. The resulting *t*_*R*_, *k*, selectivity factor (α), separation factor (*R*_*s*_), tailing factor (*TF*), and theoretical plate number (*N*) values were calculated^31,32^ to affirm system performance in line with US Pharmacopeia (Table S1).^33^

### 2.8 Studies on Determining the Linear Range

To determine the linearity range of the optimized method under optimal chromatographic conditions, a calibration study was conducted by preparing standard melatonin solutions in the concentration range of 2.0–8.0 μg/mL. For this purpose, the average peak areas obtained at the studied concentration values were used. The DPV responses were recorded for melatonin solutions over a linear concentration range of 1.9–139.4 μg/mL in 0.04 M BRB at a scan rate of 100 mV s^–1^. The regression equation and correlation coefficient (*r*) were determined. The sensitivity of the developed method was determined by the limit of detection (LOD) and limit of quantification (LOQ).^27^

### 2.9 Determination of Precision and Accuracy

Intraday and interday precision were evaluated by five replicate analyses at two concentration levels of melatonin (low and high) for chromatographic measurements and one concentration level of melatonin for voltammetric measurements. Intraday measurements were performed on the same day, and interday measurements were performed on three consecutive days. Precision was evaluated using RSD%, and accuracy was determined using Bias (%).^27^

### 2.10 Method Robustness

The robustness of the developed method was determined by examining changes in the amount of EtOH in the mobile phase (±5%, v/v), pH of the mobile phase (±0.5), flow rate (±0.2), and SDS concentration (±0.02) in the melatonin analysis. The effects of these modifications on *t*_*R*_ and peak area were determined.^27^

### 2.11 Greenness and Whiteness Evaluation of the Optimum Analysis Method

The environmental criteria of the developed methods, their compliance with the 12 principles of GAC, the Analytical Greenness Measurement (AGREE), the Green Analytical Procedure Index (GAPI), the Analytical Green Star Area (AGSA), and the Carbon Footprint Reduction Index (CaFRI) were evaluated; their compliance with the WAC approach was assessed using the Blue Applicability grade Index (BAGI) and Red Analytical Performance Index (RAPI) tools. A new tool integrating WAC and GAC principles, the Environmental, Performance and Practicability Index (EPPI), has also been applied.^9,11,34–39^

## 3 Results and Discussion

In this section, the analytical performances of the developed HMLC and DPV methods were evaluated. Initially, the optimization, validation, greenness and whiteness assessment results for each technique are presented, respectively. Finally, a statistical comparison between the two analytical methods is provided to demonstrate their efficiency and applicability for the determination of melatonin in pharmaceutical formulations.

### 3.1 Determination of Optimum Chromatographic Conditions

The analytical performance of the developed HMLC method was systematically evaluated to ensure its suitability for quantifying melatonin in formulations. The p*K*_a_ values of melatonin with indole and amid functional groups are 5.772 ± 0.001 and 10.201 ± 0.024, respectively.^40^ In this study, the pH of the mobile phase to be studied was chosen by keeping the p*K*_a_ of melatonin in the range of ±1.5 and the determined optimum EtOH amount constant. Since melatonin is in the diprotic acid form, it is retained more in the column at pH 7.0 and less at pH 8.0. Therefore, pH 7.5 was determined as the optimum pH.

Melatonin has moderate water solubility (1.07 mg/mL);^41^ therefore, the retention behavior of the compound in hydroorganic mixtures of EtOH-water was determined. Furthermore, the hydrophobicity (φ_0_) of melatonin was determined using a linear relationship between the logk values calculated for mobile phases containing 15–25% (v/v) EtOH at a constant pH (7.50) and the amount of organic solvent (φ). The calculated φ_0_ value is given in Table S2.^26,32,42^

In the analyses performed, the Ascentis RP Amide (150 × 4.6 mm, 5 μm) column, consisting of polar groups and fused core particles, was preferred instead of classical alkyl chain columns such as C18 and C8 to prevent excessive interaction of melatonin with the HPLC column. Furthermore, SDS was added to the mobile phase to modify the amide column. The added surfactant monomers strongly adsorbed onto the stationary phase, forming charged/neutral layers. The SDS-modified stationary phase acquired a net negative charge, affecting the interaction of melatonin species depending on their ionization states. While the ionic species of melatonin (HA^–^, A^2–^) interact less with the negatively charged stationary phase, the neutral form (HA) interacts more electrostatically with the stationary phase, directly affecting the attachment behavior of melatonin. In the HMLC method using both SDS and EtOH, the high concentration of organic solvent reduces or eliminates the micelle-modifier role.^43,44^ The *t*_*R*_ and *k* values of melatonin in binary mixtures containing constant concentration (0.05 M) SDS and 15%, 20%, and 25% (v/v) EtOH were determined by triplicate analysis at 37 °C and constant flow rate. Accordingly, the *k* values of melatonin in EtOH-water hydroorganic mixtures containing 15%, 20%, and 25% (v/v) EtOH were calculated as 2.870, 1.383, and 0.730, respectively. In chromatographic analyses, the shortest analysis time and the appropriate condition for the *k* value, which should be in the range of 1 < *k* < 10, were determined to be the binary mixture containing 20% (v/v) EtOH. Furthermore, 20% (v/v) EtOH was preferred in the mobile phase to maintain micelle integrity.^16,45^

### 3.2 Optimization of Chromatographic Separation and Electrochemical Conditions

In this study, the quantitative determination of melatonin in commercial tablet formulations was performed using selected IS methylparaben. In quantitative studies, compounds must have a *k* value ≥ 1, a selectivity factor (α) value ≥ 1.15, and a separation factor (*R*_*s*_) value ≥ 2. Under these conditions, an EtOH-water binary mixture containing 20% (v/v) EtOH adjusted to pH 7.5 was selected. Chromatographic parameter values calculated using the Purnell eq (Table S1)^32^ under the determined optimum chromatographic conditions are presented in Table S3. The lipophilicity values of melatonin and methylparaben are 1.63 and 1.98, respectively.^41^ These results confirm that the elution order reflects the hydrophobicity differences between melatonin and methylparaben.

The electrochemical behavior of melatonin was investigated using an unmodified gold electrode to develop a sensitive and rapid voltammetric method. The operating parameters of the DPV method were optimized before analysis and set as follows: A modulation duration of 50 ms, a modulation amplitude of 50 mV, an interval duration of 200 ms, and a step potential of 3 mV were applied.

#### 3.2.1 Influence of Supporting Electrolyte and pH on Oxidation Behavior

The type and pH of the buffer solution are critical parameters governing the electrochemical interaction between melatonin and the electrode surface. Accordingly, the influence of the supporting electrolyte and pH on melatonin oxidation was systematically investigated over the pH range of 4.0–9.0 using AB, PB, and BRB solutions by DPV.

Figure 1 presents the effects of buffer type and pH on the anodic peak current (*I*_p_) and peak potential (*E*_p_) of melatonin. Among the buffer systems examined BRB solution at pH 5.0 produced the highest *I*_p_ and the most symmetric voltammetric responses in DPV measurements (Figure 1A–C). Therefore, BRB at pH 5.0 was selected as the optimal supporting electrolyte for subsequent melatonin determination studies. In addition, the observed shift of the oxidation peak toward lower potentials with increasing pH indicates that melatonin oxidation becomes more favorable under alkaline conditions.

**Figure 1:**
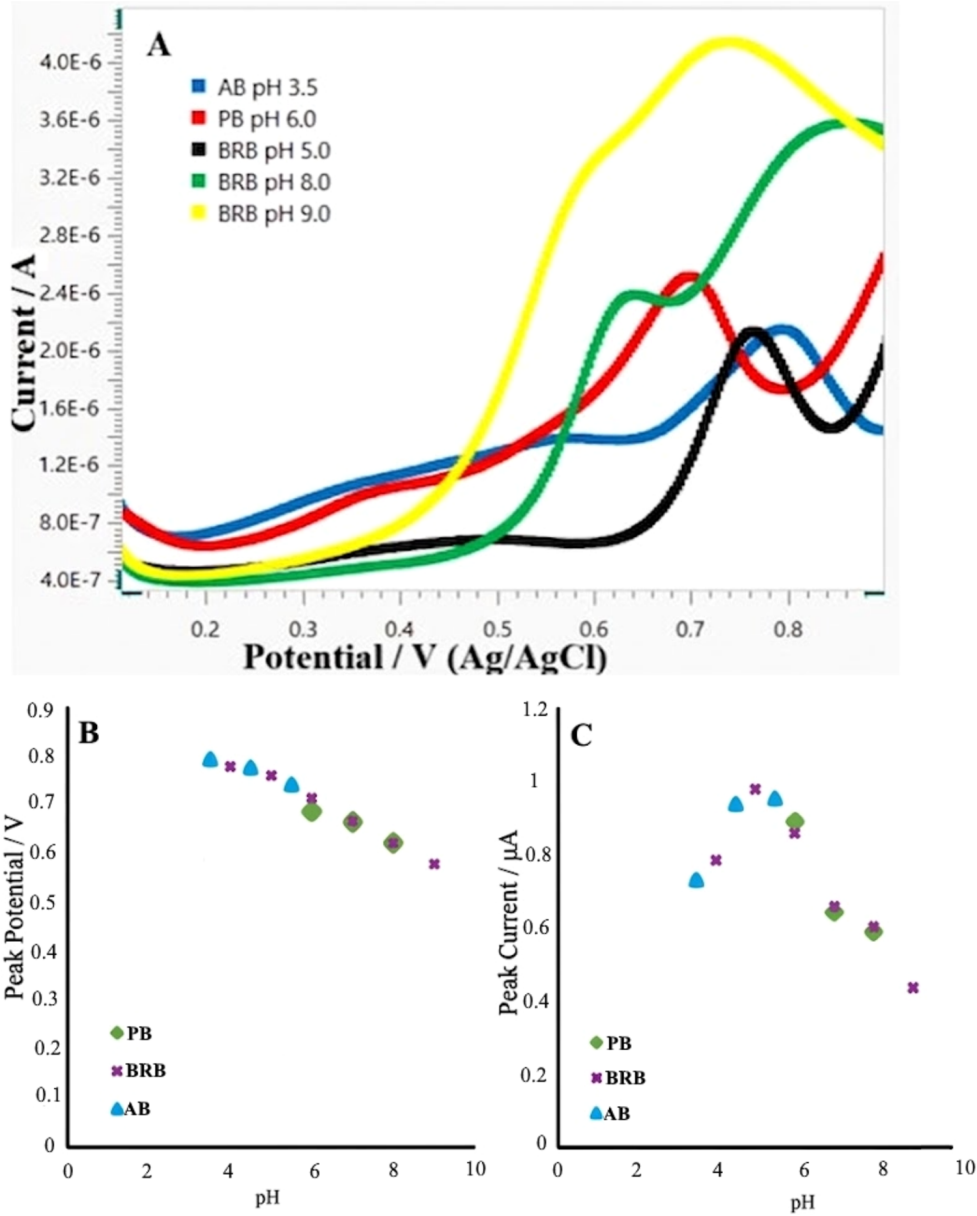
Effect of pH on the differential pulse voltammograms of 100 μg/mL melatonin (A), peak potential (B), and peak current (C).

A linear relationship between the *E*_p_ and pH was observed over the pH range of 4.0–9.0, yielding a slope of – 41.4 mV/pH with a high correlation coefficient (*r* = 0.994) in DPV measurements, and the corresponding regression equation is given in eq 1. The close agreement between the experimental slope and the theoretical value of 59/2 mV/pH indicates that the rate-determining step involves the transfer of two electrons and one proton.^46^

1 $$E_{p}\left(\mathrm{mV}\right)=955.5-41.4\;\mathrm{pH}$$

#### 3.2.2 Evaluation of Scan Rate and Electrochemical Oxidation Mechanism

The dependence of the *I*_p_ of melatonin on the scan rate was systematically evaluated using cyclic voltammetry (CV) at scan rates ranging from 50 to 250 mV/s in Britton–Robinson buffer at pH 5.0 containing 23.23 μg/mL melatonin at the gold electrode.

The *I*_*p*_ showed a linear relationship with the square root of the scan rate (v^1/2^), indicating that the electrochemical oxidation of melatonin is diffusion controlled (eq 2). This result is also supported by the linear dependence of the log *I*_*p*_ on the logarithm of the scan rate (log *v*), which produced a slope of 0.49, fairly close to the theoretical value of 0.5 expected for a diffusion-controlled process (eq 3). The agreement between these findings clearly shows that melatonin oxidation occurs predominantly under diffusion control.

2 $$I_{p}\left(\mathrm{\mu A}\right)=0.012\;v^{1/2}\left(\mathrm{mV}\;s^{-1}\right)+0.004\left(r=0.998\right)$$

3 $$\log I_{p}\left(\mathrm{\mu A}\right)=0.49\log v\left(\mathrm{mV}\;s^{-1}\right)-1.863\left(r=0.998\right)$$

### 3.3 Analytical Performance and Method Validation

#### 3.3.1 System Suitability and Chromatographic Performance

System suitability parameters were examined to evaluate the efficiency and reproducibility of the method.^33^ The *t*_*R*_ values (4.123–7.249) of the compounds indicate proper separation. The tailing factor values for both analytes were below the recommended threshold (≤2), indicating symmetrical peaks. The *N* value for melatonin (2154) and IS (3370) exceeded the required minimum of 2000, demonstrating good column efficiency. To calculate the *k* value of the compounds, the uracil solution was analyzed in triplicate (*t*_0_: 1.848 min). The *k* value calculated using the average *t*_0_ value was found to be above 1 for both compounds. Since the *R*_*s*_ value between the peaks is ≥2, it provides sufficient separation. The RSD% value, indicating reproducibility in injections for peak area and *t*_*R*_ values of the compounds, is less than 2%. The standard mixture chromatogram showing the separation of compounds under optimum chromatographic conditions is given in Figure 2. The two compounds were separated with good peak sharpness and symmetry in a total of 7.5 min.

**Figure 2:**
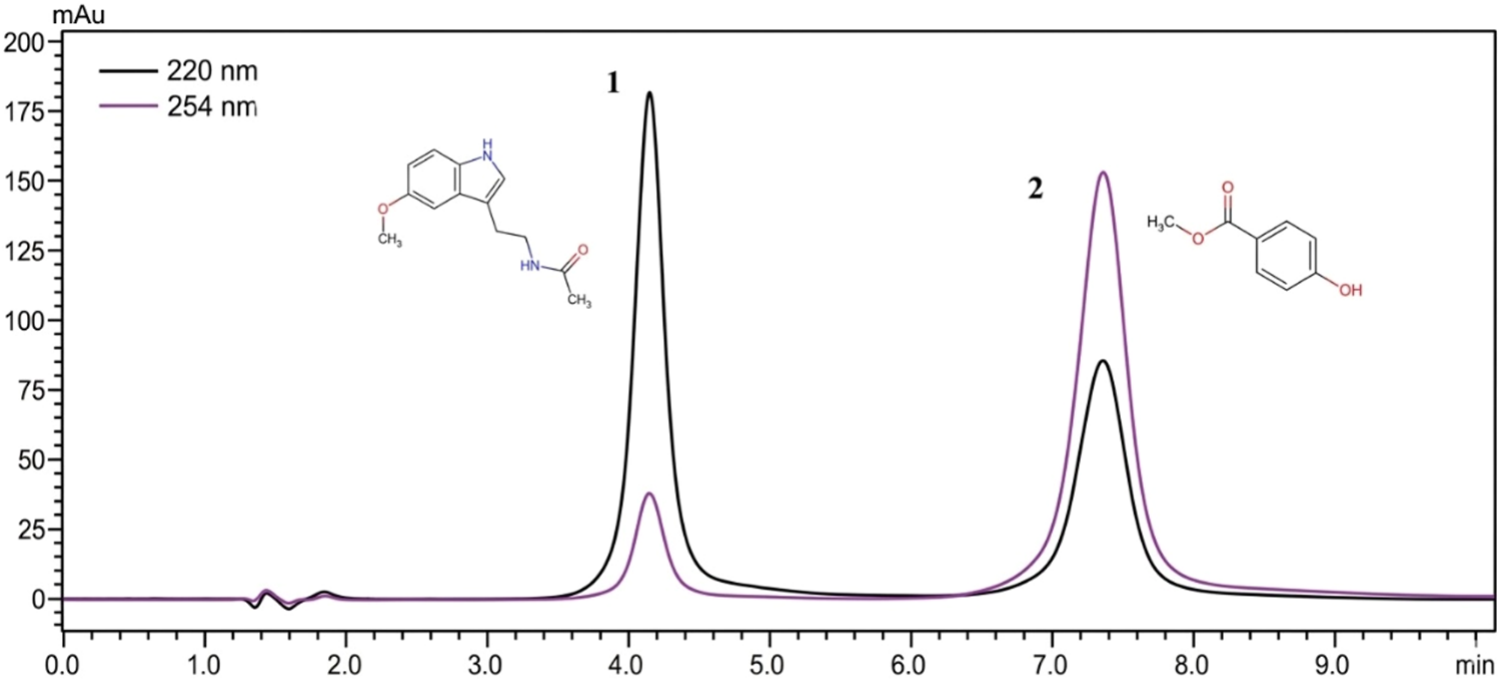
Chromatogram of the standard mixture. 1. melatonin (5.0 μg/mL) 2. Methylparaben (2.0 μg/mL).

#### 3.3.2 Linearity and Sensitivity

Method validation was carried out according to ICH Q2 (R2) guidelines.^27^ In this study, peak area ratio values obtained from melatonin and IS assays for HMLC quantitative determination were plotted against varying melatonin concentrations to derive a regression equation. Good linearity was achieved in the concentration range of 2.0–8.0 μg/mL (*r* ≥ 0.999) (Figure S1). DPV responses were recorded at a scan rate of 100 mV s^–1^ for melatonin solutions in the concentration range of 1.9–139.4 μg/mL, achieving excellent linearity (Figure 3). In electrochemical detection, linear responses are generally constrained by factors such as electrode surface saturation, mass transport limitations, and the adsorption of the analyte or its oxidation products onto the electrode surface. However, under the experimental conditions employed in the present study, these limiting factors appear to be effectively mitigated. This can be attributed to the diffusion-controlled nature of the proposed method, as confirmed by scan rate studies. In addition, the use of a bare electrode allows for both mechanical and electrochemical cleaning prior to each measurement, ensuring a reproducible and continuously renewed electrode surface while minimizing fouling and adsorption effects.

**Figure 3:**
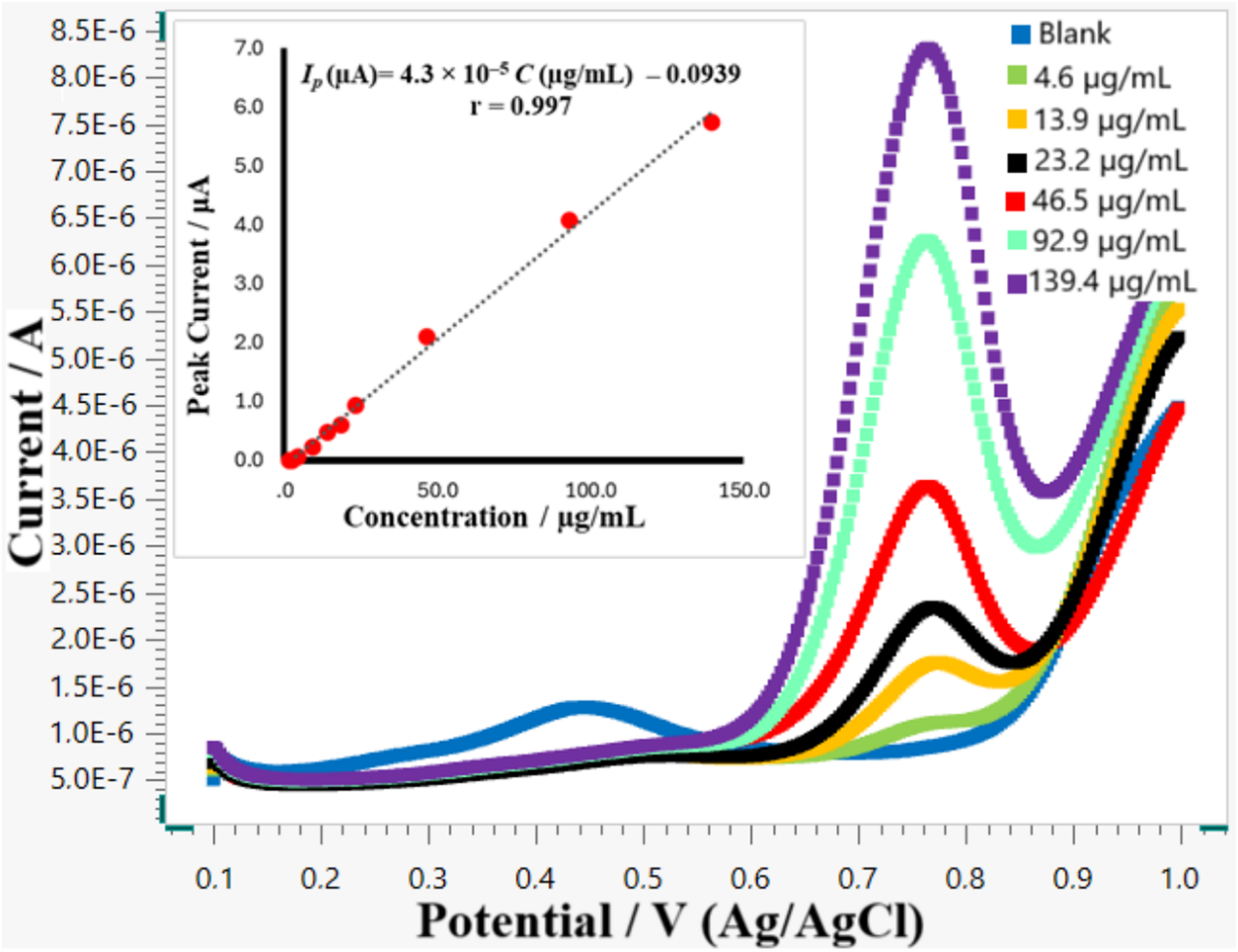
Differential pulse voltammograms of melatonin in 0.04 M BRB solution at pH 5.0 at gold electrode.

The p-values calculated from the ANOVA analysis performed on the calibration line are statistically significant for the linearity parameters. For the linear calibration range determined for the HMLC and DPV methods, the p-values for the slope were calculated as 7.78 × 10^–8^ and 0.0133, respectively. This confirms that the slope value in the calibration function is statistically different from zero and that there is significant linearity between concentration and response factor. Similarly, the *p*-values for the intercept determined for the HMLC and DPV methods were determined as 0.852 and 0.208. This is not statistically significant and supports the idea that the points where the curves intersect the *y*-axis are not different from zero. This evaluation demonstrates strong linearity in melatonin determination for both methods and reveals that quantitative analysis can be performed using the developed method.

LOD and LOQ values for melatonin were determined according to signal-to-noise ratios of 3.3:1 and 10:1, respectively. LOD values were calculated as 0.335 μg/mL and 0.481 μg/mL for HMLC and DPV methods, respectively; LOQ values were calculated as 1.016 μg/mL and 1.456 μg/mL. Low LOD and LOQ values indicate that the developed method has good sensitivity.

#### 3.3.3 Precision Data of Methods

Intraday and interday studies were conducted to determine the reproducibility of the developed methods. For the HMLC study, five replicate analyses were performed using melatonin solutions prepared at concentrations of 4.0 μg/mL and 7.0 μg/mL within the linear working range, and a constant concentration of IS. Concentration and RSD% values calculated using peak area ratio values are presented in Table 1. The results obtained demonstrate the high reproducibility (RSD%) and accuracy (Bias%) of this developed method under routine laboratory conditions.

**Table 1:** Intra-Day and Inter-Day Results for HMLC Method

	Intraday			Interday		
Theoretical concentration (μg/mL)	Mean concentration (±SD)	RSD%	Bias%	Mean concentration (±SD)	RSD%	Bias%
4	4.061 ± 0.026	0.629	1.521	4.041 ± 0.041	1.009	1.031
7	7.093 ± 0.046	0.641	1.331	7.130 ± 0.072	1.005	1.858

Similarly, the accuracy of the method was evaluated by performing five repeated measurements using at least three independently prepared solutions at the same melatonin concentration (46.46 μg/mL). Both intraday and interday accuracy were assessed over a one-week period. Intraday and interday RSD% values were calculated as 0.842% and 0.907%, respectively. These results indicate that the precision of the method is also good.

#### 3.3.4 Accuracy Data of Methods

The accuracy of the developed DPV method was evaluated through comprehensive recovery studies. These experiments aimed to determine the potential matrix interference of common pharmaceutical excipients on the quantification of melatonin. Initially, the accuracy of the proposed method was assessed by comparing the labeled content of melatonin in commercial tablets against the experimentally derived values obtained from the regression equation.

In the HMLC study, a standard melatonin solution (3 μg/mL) was prepared within the calibration range and added to a tablet sample containing the active ingredient melatonin (Ocean, Orzax, 3 mg Melatonin). Similarly, for the DPV study, a 46.46 μg/mL standard melatonin solution was added to the tablet sample. The amount of melatonin in the sample was determined by monitoring changes in peak current for DPV using peak area ratio values obtained from HPLC analysis. Using these results, the recovery values of the methods could be calculated (Table 2).

**Table 2:** Recovery Results of Melatonin Tablets Analyzed Using the Developed Method

Parameters	HMLC	DPV
Labeled Claim (mg)	3.000	3.000
Found Amount (mg) (Mean ± SD)	2.987 ± 0.051	3.052 ± 0.018
RSD (%)	1.714	0.590
Bias (%)	–0.433	1.733
Recovery (%) (Mean ± SD)	99.570 ± 1.754	100.39 ± 0.934
RSD (%)	1.722	0.590
Bias (%)	–0.430	0.390

According to the ICH guidelines, the average recovery should be between 95% and 105%.^27^ This result demonstrates the high accuracy of the method. In this quantitative analysis study, the excipients commonly used in tablets did not affect the analysis results. Chromatograms showing the tablet sample and the tablet sample with a specific concentration of melatonin added for the recovery study are given in Figure 4. No interference peaks were observed in the chromatograms.

**Figure 4:**
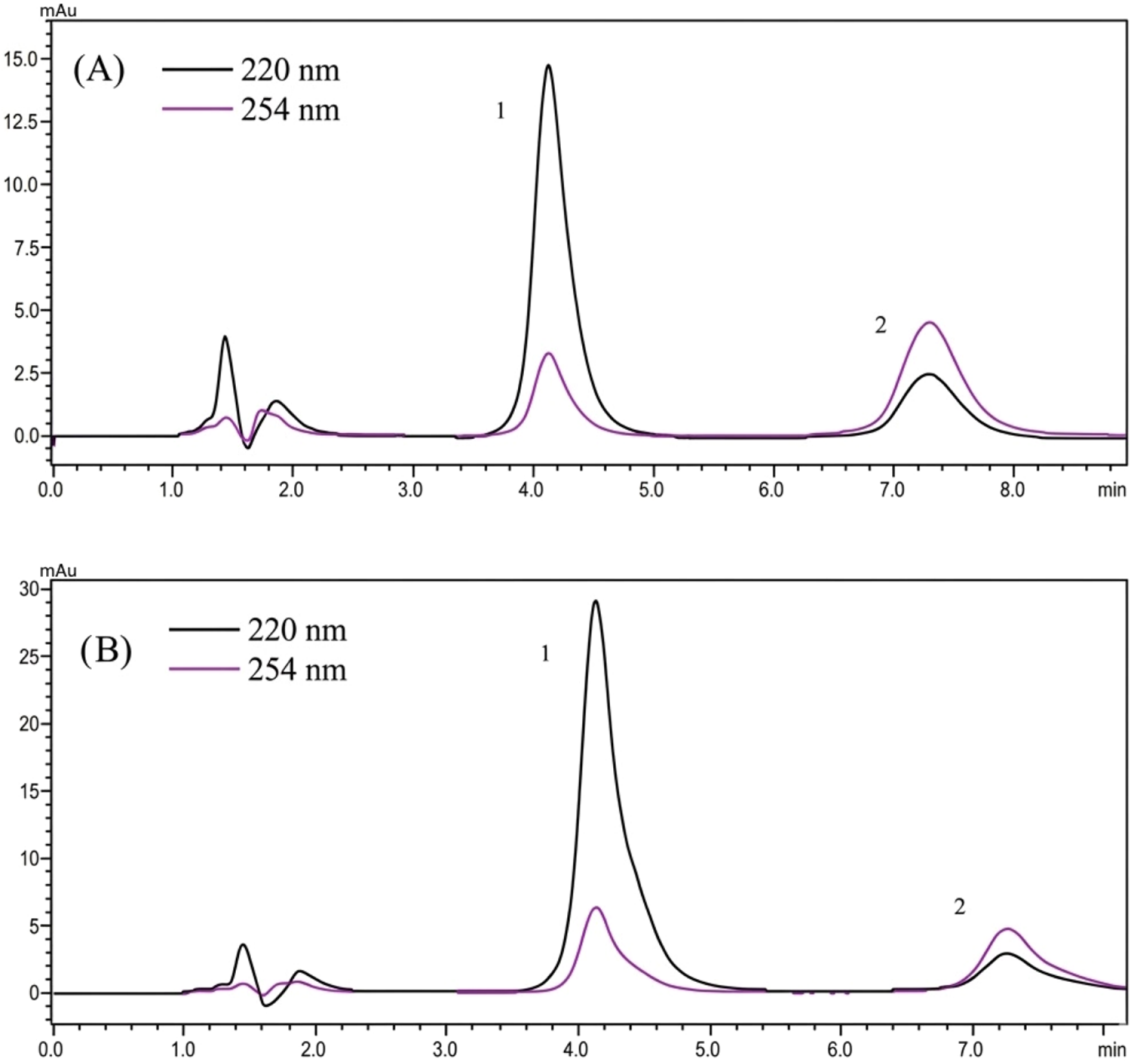
(A) Tablet sample analysis (1.melatonin 3.0 μg/mL; 2. methylparaben 2.0 μg/mL) (B) Spiked sample analysis (1.melatonin 6.0 μg/mL; 2.methylparaben 2.0 μg/mL).

#### 3.3.5 Robustness Test

Robustness testing is crucial in method validation to ensure that small changes in experimental conditions do not affect the reliability of the analytical method.^27^ *t*_*R*_ and peak area were monitored under each condition. The results showed that it affected the retention time but did not significantly affect the peak areas, confirming the robustness of the method under small changes in chromatographic conditions (Table 3).

**Table 3:** Robustness Data of the Method under Varying Chromatographic Conditions

Condition	*t*_*R*_ (0.8)^a^	*t*_*R*_ (1.0)	*t*_*R*_ (1.2)	Area (0.8)	Area (1.0)	Area (1.2)
pH 7.5–0.05 M SDS (20% EtOH)	5.265	4.123	3.834	1239117	1216147	1183884
pH 7.5–0.05 M SDS (15% EtOH)	6.892	5.520	4.606	1274073	1193369	1076296
pH 7.5 −0.03 M SDS (20% EtOH)	5.842	4.681	3.909	1392688	1285562	1115467
pH 7.0–0.05 M SDS (20% EtOH)	5.544	5.092	4.419	1338574	1283470	1067343
pH 8.0–0.05 M SDS (20% EtOH)	5.012	4.047	3.385	1338399	1256241	1071221
pH 7.5–0.07 M SDS (20% EtOH)	4.875	3.887	3.252	1146386	1080993	968770
pH 7.5–0.05 M SDS (25% EtOH)	3.081	2.279	1.712	1307486	1283490	1048915

a Mobile phase flow rate (mL/min).

### 3.4 Comparison of HMLC and DPV Methods

The main objective of this study is to evaluate and compare the selectivity, sensitivity, and applicability performance of these methods for the determination of melatonin in pharmaceutical dosage forms for rapid screening and quality process control. The results obtained for pharmaceutical dosage forms using two different analytical techniques, DPV and HPLC, were statistically compared. At a 95% confidence level, the F and t values calculated from the recovery data were 0.29 and 0.36, respectively. These values are significantly lower than the corresponding critical values, indicating that there is no statistically significant difference between the DPV and HPLC results obtained for pharmaceutical dosage forms.

Although HMLC is an attractive analytical approach due to its reduced solvent consumption, it has some disadvantages compared to conventional HPLC studies, such as limited chromatographic efficiency and relatively poor elution power, especially for strongly hydrophobic analytes or complex matrices, despite its advantages in green analytical chemistry applications.^47^ Furthermore, the analytical performance of HMLC methods largely depends on the careful optimization of various experimental parameters, including surfactant concentration, organic modifier content, pH, and ionic strength; this can make routine application difficult.^48^ However, literature studies show that in the analysis of ionizable compounds, the HMLC method is superior to conventional HPLC in terms of short time, high separation factor, and cost.^19,44^

Although HPLC provides high sensitivity and selectivity, it typically involves labor-intensive sample preparation, considerable solvent consumption, and costly instrumentation, making the overall process more time-consuming, expensive, and complex. In contrast, electrochemical techniques offer notable advantages, including high sensitivity, low cost, good stability, and fast response times.^49^ DPV has advantages such as low cost, short analysis time, operational simplicity, and relatively low sensitivity to environmental variations like temperature and pH changes.^50^

A large number of electrochemical methods have been reported in the literature for the analysis of melatonin.^8^ However, most of these methods employ modified electrodes, which are time-consuming to prepare, costly, and may lead to stability issues. In contrast, Bare electrodes, on the other hand, do not require modifications, making them simpler, more cost-effective, more reproducible, and stable, while also allowing easy surface cleaning and facilitating method transfer between laboratories.^51^ In addition, the absence of a modification step reduces both waste generation and chemical consumption, thereby enhancing the environmental sustainability and green character of the method.^52^ Furthermore, the ability to perform analyses at high concentrations within the broad calibration range achieved in our study is attributed to the mechanical and electrochemical cleaning of the electrode surface after each measurement, which prevents surface fouling or degradation, unlike modified electrodes.^53,54^ Compared to the studies reported in the literature, the capacity to analyze higher concentrations also represents an additional advantage of our work. Furthermore, the analytical performance of the proposed method is comparable to those reported in the literature^55–57^ and in some cases even superior.^58^ It is worth noting that although certain studies have achieved lower LOD values and/or wider linear ranges,^59–63^ these approaches generally rely on complex, time-consuming, and costly electrode modification procedures, which often involve nonsustainable and environmentally unfriendly processes. In this context, the DPV method developed using a bare gold electrode emerges as a compelling alternative due to its simplicity, rapid applicability, and cost-effectiveness, while still maintaining satisfactory analytical performance.

More specifically, although the method reported by El-Sayed et al.^55^ exhibits a lower LOD compared to the present study, the proposed method offers a significantly wider linear range. In the study by Sunon et al.,^56^ while the modified SPCE/Chi–CeO_2_ electrode provides a lower LOD than the bare SPCE, the method developed in this work outperforms the bare SPCE in all aspects and still offers a broader linear range than the modified system. Similarly, although a lower LOD is reported by Martínez-Moro et al.,^57^ that study employs a modified electrode, whereas the present method achieves competitive sensitivity alongside a wider linear range without requiring any modification step. In addition, when compared to Bekheit et al.,^58^ the proposed method demonstrates clear superiority in terms of both LOD and linear range.

Overall, these findings demonstrate that the proposed simple, sensitive, and accurate method, achieved without any electrode modification, holds significant promise for reliable melatonin determination.

Many studies have been conducted on the determination of melatonin using various HPLC methods, but most of them are limited due to the use of toxic solvents or long analysis times.^5,64–66^ Sezgin et al. developed green methods for melatonin determination using EtOH-water binary mixtures and different detectors. Using photodiode array (PDA) and fluorescence detectors, they achieved determination at lower concentrations (ng/mL) compared to our study.^67^ As with other HPLC methods, a lower concentration of EtOH was used in our study (Table S4). Although melatonin is an ionizing compound, pH control in the mobile phase has not been achieved in most studies. One of the most important advantages of the method developed in this study is that pH standardization suitable for the water-organic solvent binary mixture is achieved, rather than electrode calibration in an aqueous environment. The limitation of the EtOH solution used in our study is due to the use of SDS. Importantly, unlike the existing literature which focuses only on liquid chromatography, this study provides a faster screening alternative by determining with an electrochemical method, thus providing a rapid analysis method alternative.

### 3.5 Ecological Impact Assessment

This work presents a comprehensive evaluation of two different analytical methods (HMLC and DPV) developed for melatonin determination, in terms of environmental sustainability and performance. The study combines the principles of GAC and WAC to determine melatonin content in pharmaceutical tablets, offering an assessment of cost, efficacy, and reliability. It is the first in the literature to evaluate both HMLC and DPV methods together.

The accuracy and environmental impact of the methods were analyzed using various complementary metrics. Compliance with 12 core principles was measured using AGREE, GAPI, and AGSA tools for greenness and sustainability.^9,11,34^ To evaluate the effectiveness of carbon footprint reduction initiatives adopted in the analytical method development process, the innovative CaFRI tool, which measures greenhouse gas emissions, was used, unlike classical tools.^35^ The EPPI index, which combines GAC, WAC, and Green Sample Preparation (GSP) principles in a single structure, and the BAGI and RAPI tools for WAC compliance were used to evaluate the methods.^36–39^

The AGREE tool is shown as a pictogram in red, yellow, and green, arranged clockwise. The closer the resulting score is to 1, the more environmentally friendly the method is. This scale is correlated with a color scale from dark green to dark red. The appropriately colored middle digit in the AGREE pictogram represents the final average numerical value obtained from the 12 data points. The range of the color map used in the graph and the corresponding values are given in Figure S1. The performance of the procedure in each evaluation criterion is reflected by the color in the segment where the number corresponding to each criterion is located. In Figure 5, the AGREE software score of the DPV method is higher than that of HMLC, indicating that it is more environmentally friendly.^9^

**Figure 5:**
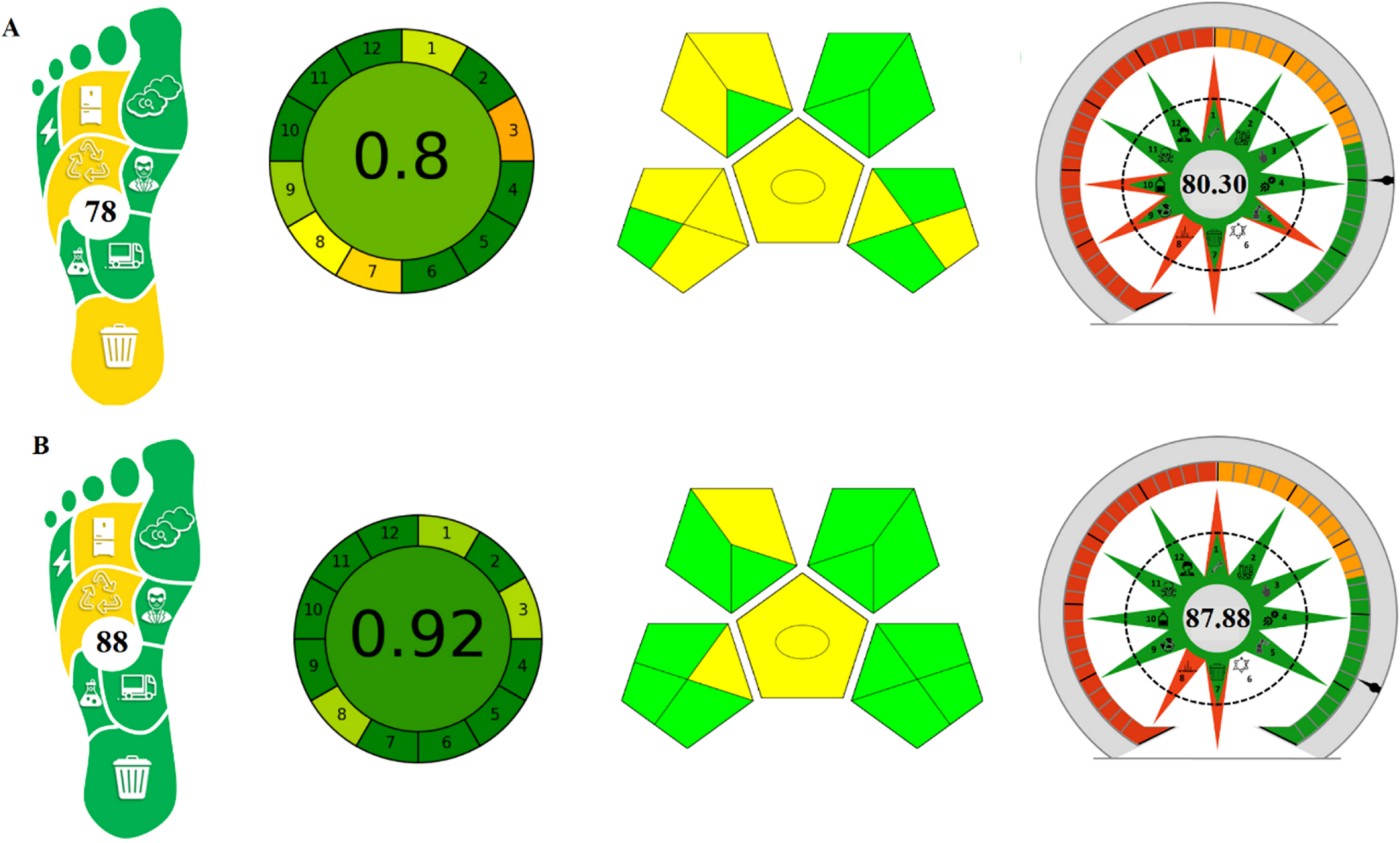
Evaluation of methods with green tools (A) HMLC (B) DPV methods.

GAPI is a semiquantitative tool consisting of five pentagrams. In GAPI, a green, yellow, and red color scale is used to indicate the low, medium, and high environmental impact of analytical procedures, respectively.^11^ The main features and 15 criteria of GAPI are shown in Figure S2.^68^ The evaluation using the GAPI tool shows that neither method contains red in the pentagrams. The DPV method is environmentally friendly due to its lower energy, solvent, and waste consumption compared to the HMLC method Figure 5.

AGSA is a tool specifically designed to evaluate analytical methods according to the 12 principles of GAC, developed by Mansour et al.^34^ The AGSA pictogram encompasses the 12 principles, each evaluated through specific questions with three-level response options, allowing for a hierarchical differentiation among methods (Figure S2). Higher scores indicate greater sustainability and support strategies involving minimal sample processing, low energy consumption, nonhazardous reagents, and waste minimization. These scores are cumulative, and a total of 36 points (12 principles × 3 points) represents 100%.^34^ Ecological impact is given as an evaluation score in the pictogram, from highest (dark green) to lowest (dark red). The AGSA metric, which provides quantitative and visual information, offers a holistic assessment. The DPV method, like other tools, showed a high degree of greenness with the AGSA result of 87.88% (Figure 5).

Although there are many tools for assessing greenness in analytical methods, the carbon footprint is not considered in the applications used. Therefore, in this study, the Carbon Footprint Reduction Index (CaFRI) was used to evaluate the sustainability and greenhouse gas emissions of liquid chromatographic methods developed in hydroorganic solvent environments.^35^ This tool provides an assessment of environmental sustainability with parameters related to greenhouse gas emissions. This assessment is a complement to greenness assessment tools. The CaFRI, shown as a pictogram in the shape of a human foot, uses red, yellow, and green colors to indicate low, average, and green ratings, respectively (Figure S2). The carbon footprint of methods developed for the determination of melatonin has been evaluated. As shown in Figure 5, the DPV method, which has a higher numerical score (score 88), is more environmentally friendly than the HMLC method (score 78). This is because the HMLC method requires more energy-intensive use and expert analysts. To improve the environmentally friendly performance of the method, it may be necessary to reduce CO_2_ emissions and implement waste recycling.

In recent years, the WAC approach has also been used as an alternative to assess the greenness of methods.^36^ WAC evaluates the quality, environmental impact, and economic feasibility of a method using red and blue colors in addition to green. The RAPI tool has been used to assess the ″redness″ of analytical methods by focusing on ten key analytical parameters. RAPI is used for quantitative analysis methods.^37^ In this study, an evaluation was performed using RAPI according to the 10 key validation parameters of the ICH guideline under optimum analysis conditions. The sum of all scores in the range of 0–100 is placed in the middle part of the pictogram (Figure S2). The better the overall performance of the method, the higher this value. According to this tool, a score between 75 and 89 indicates that the analytical method is suitable for many applications (Figure 6). Accordingly, the DPV method is more applicable than the HMLC method.

**Figure 6:**
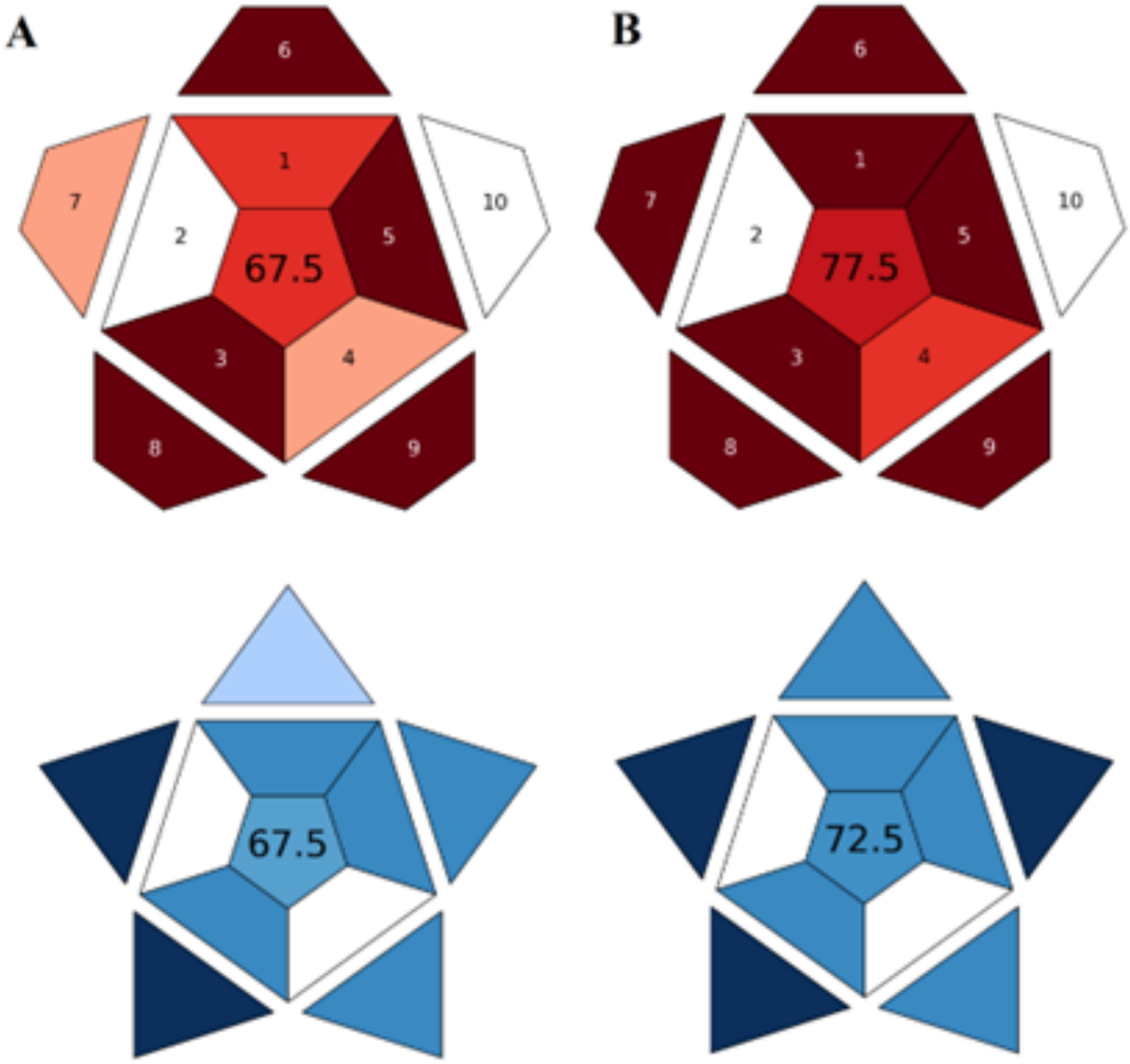
Evaluation using RAPI and BAGI tools. (A) HMLC (B) DPV methods.

Unlike traditional green criteria that focus on environmental impact, BAGI evaluates various factors important for the applicability of the analytical method.^38^ The BAGI tool provides two types of results: a score and an asteroid pictogram. The pictogram is divided into ten sections representing performance criteria (Figure S2). Figure 6 shows the BAGI pictograms for both methods. The color gradient of the pictogram indicates how well the method meets the defined criteria: dark blue indicates high compliance, blue indicates medium compliance, light blue indicates low compliance, and white indicates noncompliance. In the middle area, a total score ranging from 25 to 100 is shown; 25 indicates poor performance, and 100 indicates excellent performance.^69^ Analytical methods scoring above 60 on the BAGI pictogram are considered practical. Accordingly, the methods developed in this study are practical in terms of applicability.

A comprehensive evaluation of both methods was also carried out with the newly developed EPPI, which integrates the principles of GAC, GSP and WAC.^39^ EPPI is evaluated with two subindices. The Environmental Impact Index (EI) indicates the greenness of the method, while the Performance and Practicality Index (PPI) shows performance based on time, cost, accuracy, validation, and resource efficiency. The total EPPI score is calculated by combining the EI and PPI, each contributing 50% to the overall score (Figure 7). Accordingly, the EPPI index scores for DPV and HMLC were calculated as 86.1 and 79.4, respectively.

**Figure 7:**
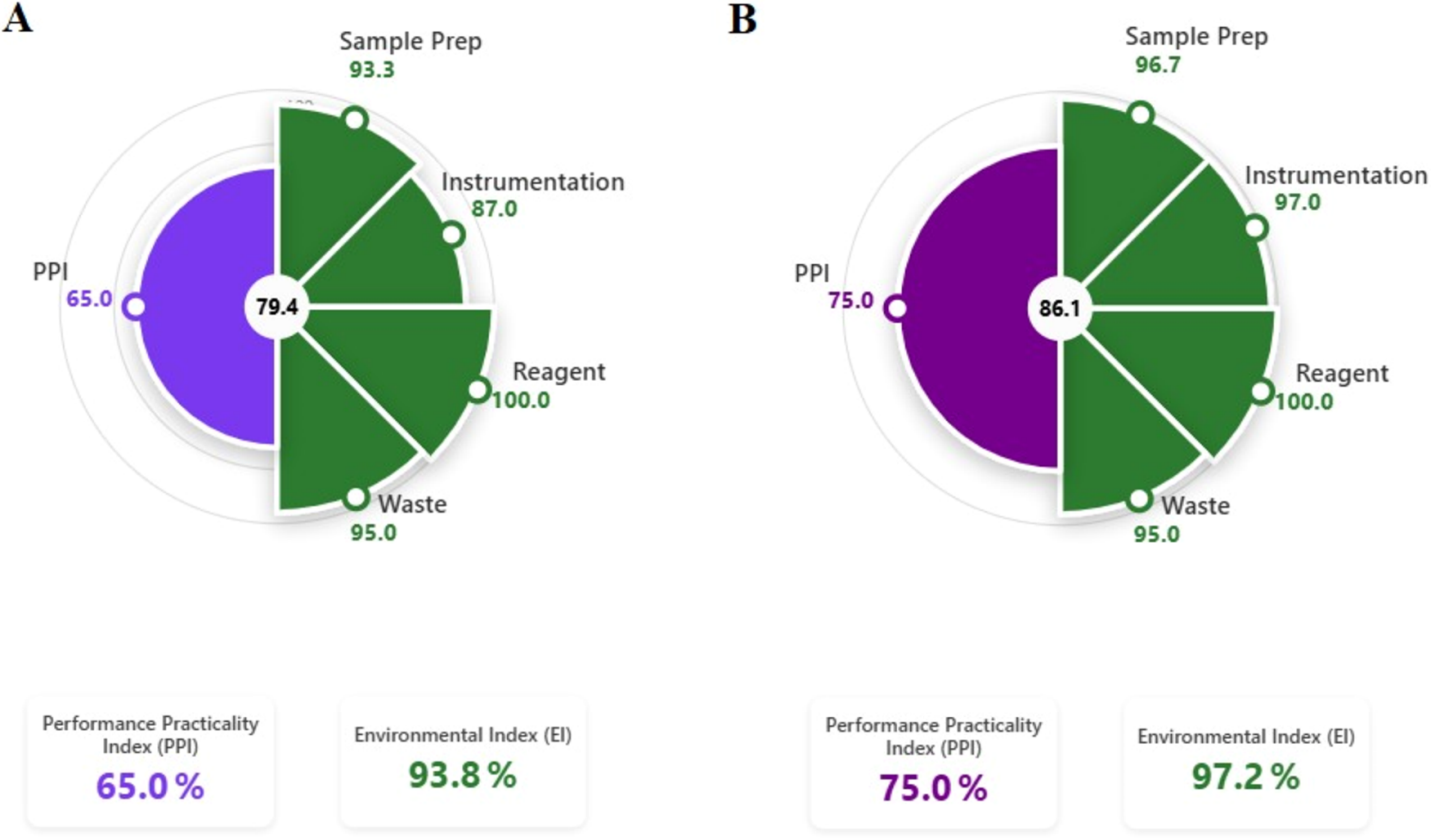
Graphical representation of the EPPI index: (A) HMLC, (B) DPV methods.

The EPPI index presented in Figure 7 is a pie chart, with each method represented by two adjacent halves. The value in the middle of the graph gives the total score. Accordingly, since both methods are between 75 and 100 points, they yielded green and practical, efficient method results.

## 4 Conclusions

This study provides quantitative benchmarks for method optimization in melatonin determination in pharmaceutical tablets using both HMLC and DPV methods. The developed HMLC method eliminates the need for toxic solvents by using an EtOH-based mobile phase and SDS, while the DPV method provides fast and sensitive screening with a gold electrode. Method validation showed excellent results in terms of linearity, sensitivity, accuracy, precision, LOD and LOQ parameters, and robustness. An analytical performance comparison using *t-* and *F-*tests at a 95% confidence level showed no significant differences between the results. This comprehensive study is the first to use green and miniaturized methods for the determination of melatonin. While both green, white, sustainability, and applicability methods received good scores, the DPV method proved superior to the HMLC method. Accordingly, qualitative and quantitative results for DPV were evaluated using GAPI, AGREE (0.92 points), AGSA (87.88 points), RAPI (77.5 points), CAFRI (88.0 points), BAGI (72.5 points), and EPPI (86.1 points) tools. In conclusion, these two analytical methods were successfully applied to determine the amount of melatonin in commercially available tablets in Türkiye, demonstrating the practical applicability of the methods and their potential for routine quality control analyses.

## Supplementary Material



## Data Availability

The data that support the findings of this study are available within the article.

## Notes

System suitability parameters and formulas (Table S1); data for the EtOH-log k relationship (Table S2); chromatographic performance data calculated via the Purnell equation (Table S3); performance comparison with reported HPLC methods (Table S4); calibration graph of melatonin for the RPLC method (Figure S1); and pictorial representations of the tools used for greenness and whiteness evaluation (Figure S2) (PDF)

## References

[1] Reiter R. J., Tan D. X., Rosales-Corral S., Manchester L. C. (2013). The universal
nature, unequal distribution and antioxidant functions of melatonin
and its derivatives. Mini-Rev. Med. Chem..

[2] Mayo J. C., Sainz R. M., Tan D. X., Hardeland R., Leon J. (2005). Anti-inflammatory actions
of melatonin and its metabolites,
N1-acetyl-N2-formyl-5-methoxykynuramine (AFMK) and N1-acetyl-5-methoxykynuramine
(AMK), in macrophages. J. Neuroimmunol..

[3] Hardeland R. (2018). Melatonin
and inflammation—Story of a double-edged blade. J. Pineal Res..

[4] Bubenik G. A., Konturek S. J. (2011). Melatonin and aging:
Prospects for human treatment. J. Physiol. Pharmacol..

[5] Abo
Elkheir S. M., Nasr J. J. M., Walash M. I., Zeid A. M. (2025). Eco-friendly
spectrofluorimetric and HPLC-fluorescence methods for simultaneous
determination of melatonin and zolpidem in pharmaceuticals. Sci. Rep..

[6] Alessa H., Althakafy J. T., Saber A. L. (2020). Electroanalytical and spectrophotometric
methods for the determination of melatonin-a review. Int. J. Electrochem. Sci..

[7] Apetrei I. M., Apetrei C. (2016). Voltammetric determination
of melatonin using a graphene-based
sensor in pharmaceutical products. Int. J. Nanomed..

[8] Barrera-Quiroz A., Méndez-Albores A., González-Fuentes M. A., Méndez E. (2025). Electroanalytical
sensing of melatonin and its applications
in pharmaceutics and biology. Electroanalysis.

[9] Pena-Pereira F., Wojnowski W., Tobiszewski M. (2020). AGREE—Analytical GREEnness
Metric Approach and Software. Anal. Chem..

[10] Kalkir F., Demiralay E. Ç., Daldal Y. D., Yilmaz H. (2025). Chromatographic analysis
and pKa evaluation of active pharmaceutical ingredients in anti-metastatic
breast cancer: Green vs. conventional RPLC. J. Pharm. Biomed. Anal..

[11] Płotka-Wasylka J. (2018). A new tool
for the evaluation of the analytical procedure: Green analytical procedure
index. Talanta.

[12] Öztürk Z., Çubuk
Demiralay E., Yilmaz H. (2025). Determination of Retention
Behavior and pKa Values of Some Phenothiazines with Green Chemistry
Approach. ACS Omega.

[13] Mabrouk M., Abdelfattah I. I., Mansour F. R. (2023). Green method for
determination of
four anti-viral drugs using micellar liquid chromatography: application
to dosage form analysis. Sustainable Chem. Pharm..

[14] Bahgat E. A., Khalil H., Reda A., Fawzy M. G. (2026). Micellar liquid
chromatographic method for quantitative analysis of nasal spray combinations
containing Mometasone furoate: Sustainability evaluation by GSST,
AGREE, and GAPI. Microchem. J..

[15] Cherehani R., Yadav D., Yadav R. (2025). Micellar liquid chromatography:
A
green and sustainable approach for the simultaneous pharmaceutical
drug analysis. Results Chem..

[16] López-Grío S., Baeza-Bacza J. J., García-Alvarez-Coque M. C. (1998). Influence
of the addition of modifiers on solute-micelle interaction in hybrid
micellar liquid chromatography. Chromatographia.

[17] García-Ferrer D., Peris-Vicente J., Bose D., Durgbanshi A., Carda-Broch S. (2024). Hybrid micellar liquid chromatography separation of
brimonidine tartrate and brinzolamide. Retention study and quantification
in fixed dose ophthalmic suspensions. Microchem.
J..

[18] Marie A. A., Yassin M. G., Roshdy A. (2025). Stability
indicating green micellar
liquid chromatographic method for simultaneous analysis of Metformin
and dapagliflozin in their tablets. BMC Chem..

[19] Daldal Y. D., Çubuk Demiralay E. (2025). Use of green
RPLC methods as an alternative
to conventional RPLC methods in the determination of the pKa values
of some H2 receptor antagonists. J. Liq. Chromatogr.
Relat. Technol..

[20] Siddiqui M. R., AlOthman Z. A., Rahman N. (2017). Analytical techniques
in pharmaceutical
analysis: A review. Arabian J. Chem..

[21] Uslu B., Doğan B., Özkan S. A. (2005). Electrochemical studies of ganciclovir
at glassy carbon electrodes and its direct determination in serum
and pharmaceutics by square wave and differential pulse voltammetry. Anal. Chim. Acta.

[22] Arellano M., Oturan N., Oturan M. A., Pazos M., Sanromán M. Á., Gonzalez-Romero E. (2020). Differential
pulse voltammetry as
a powerful tool to monitor the electro-Fenton process. Electrochim. Acta.

[23] Prete, M. C. ; Rocha, L. R. ; Tarley, C. R. T. Development of new electroanalytical method based on graphene oxide-modified glassy carbon electrode for mephedrone illicit drug determination. In Carbon Nanomaterials-Based Sensors; Elsevier, 2022; pp 43–56.

[24] Yun
Xia H., Xiao Y. H. (2005). Determination of isoniazid using a gold electrode by
differential pulse voltammetry. Anal. Lett..

[25] Yan X., Ma S., Tang J., Tanner D., Ulstrup J. (2021). Direct
electron transfer of fructose dehydrogenase immobilized on thiol-gold
electrodes. Electrochim. Acta.

[26] Valkó K., Slégel P. (1993). New chromatographic
hydrophobicity index (ϕ0)
based on the slope and the intercept of the log k′ versus organic
phase concentration plot. J. Chromatogr. A.

[27] ICH Q2­(R2) Guideline on Validation of Analytical Procedures 20025 https://www.ema.europa.eu/en/documents/scientific-guideline/ich-q2r2-guideline-validation-analytical-procedures-step-5-revision-1_en.pdf. (accessed Nov 10, 2025).

[28] Rondinini S., Mussini P. R., Mussini T., Vertova A. (1998). pH measurements in
non-aqueous and mixed solvents: predicting pH (PS) of potassium hydrogen
phthalate for alcohol-water mixtures (technical report). Pure Appl. Chem..

[29] Baucke F. G. K., Naumann R., Alexander-Weber C. (1993). Multiple-point calibration with linear
regression as a proposed standardization procedure for high-precision
pH measurements. Anal. Chem..

[30] Nane İ. D., Çubuk Demiralay E., Daldal Y. D. (2024). Comparison of classical
and green RPLC methods in the determination of pKa values of some
piperazine group antihistamines. Chem. - Eur.
J..

[31] Kazakevich, Y. V. ; Lobrutto, R. HPLC for Pharmaceutical Scientists; John Wiley & Sons, Inc.: Germany, 2006.

[32] Poole, C. F. ; Poole, S. K. Chromatography Today; Elsevier Science: New York, 1991.

[33] United States Pharmacopeia and National Formulary (USP 37-NF 32); United States Pharmacopeial Convention: Rockville, MD, 2014.

[34] Mansour F. R., Bedair A., Belal F., Magdy G., Locatelli M. (2025). Analytical
Green Star Area (AGSA) as a new tool to assess greenness of analytical
methods. Sustainable Chem. Pharm..

[35] Mansour F. R., Nowak P. M. (2025). Introducing the
carbon footprint reduction index (CaFRI)
as a software-supported tool for greener laboratories in chemical
analysis. BMC Chem..

[36] Pingili D., Awasthi A., Varshney M. M. (2025). White analytical
chemistry: a review
of current developments. J. Anal. Chem..

[37] Nowak P. M., Wojnowski W., Manousi N., Samanidou V., Płotka-Wasylka J. (2025). Red analytical
performance index (RAPI) and software:
the missing tool for assessing methods in terms of analytical performance. Green Chem..

[38] Manousi N., Wojnowski W., Płotka-Wasylka J., Samanidou V. (2023). Blue applicability
grade index (BAGI) and software: a new tool for the evaluation of
method practicality. Green Chem..

[39] Elagamy S. H., Kannaiah K. P., Chanduluru H. K., Yahaya N., Obaydo R. H. (2025). EPPI: A
comprehensive index framework for assessment of sustainability performance,
and practicality of analytical methods. Green
Anal. Chem..

[40] Zafra-Roldán A., Corona-Avendaño S., Montes-Sánchez R., Palomar-Pardavé M., Romero-Romo M., Ramírez-Silva M. (2018). New insights on the spectrophotometric determination
of melatonin pKa values and melatonin-βCD inclusion complex
formation constant. Spectrochim. Acta, Part
A.

[41] SwissADME . 2025, http://www.swissadme.ch/. (accessed Feb 16, 2025).

[42] Üstün Z., Demiralay E. Ç. (2025). Determination of the dissociation constants of six
pharmacologically active imidazole antifungal agents by RPLC method. Microchem. J..

[43] Berthod, A. ; García-Alvarez-Coque, M. C. Micellar Liquid Chromatography; Marcel Dekker: New York, 2000.

[44] Konçe İ., Çubuk Demiralay E. (2023). Use of green analysis
methods to
determine the ionization constant values of isoxazolyl penicillins. J. Mol. Liq..

[45] Ruiz-Angel M. J., Carda-Broch S., Torres-Lapasió J. R., Simó-Alfonso E. F., García-Alvarez-Coque M. C. (2002). Micellar-organic versus aqueous-organic
mobile phases for the screening of β-blockers. Anal. Chim. Acta.

[46] Smyth, M. R. ; Vos, J. G. Analytical Voltammetry; Elsevier Science: Netherlands, 1992.

[47] Ruiz-Ángel M. J., García-Álvarez-Coque M. C., Berthod A. (2009). New insights and recent
developments in micellar liquid chromatography. Sep. Purif. Rev..

[48] Yabré M., Ferey L., Somé I. T., Gaudin K. (2018). Greening reversed-phase
liquid chromatography methods using alternative solvents for pharmaceutical
analysis. Molecules.

[49] Öztürk G., Sofu Ü., Kul D. (2024). Development of low-cost, fast, and
sensitive voltammetric methods for the determination of donepezil
using unmodified carbon paste electrode. Monatsh.
Chem..

[50] Wang H. Y., Pan M. L., Oliver Su Y. L., Tsai S. C., Kao C. H. (2011). Comparison of Differential Pulse Voltammetry (DPV)—a new method
of carbamazepine analysis—with Fluorescence Polarization Immunoassay
(FPIA). J. Anal. Chem..

[51] Redivo L., Stredanský M., De Angelis E., Navarini L., Resmini M., Švorc Ĺ. (2018). Bare
carbon electrodes as simple and efficient sensors
for the quantification of caffeine in commercial beverages. R. Soc. Open Sci..

[52] Kiliç A., Aslan M., Levent A. (2024). Investigation of the electrochemical
properties of edoxaban using glassy carbon and boron-doped diamond
electrodes and development of an eco-friendly and cost effective voltammetric
method for its determination. Anal. Biochem..

[53] Uslu B., Ozkan S. A. (2007). Solid electrodes
in electroanalytical chemistry: present
applications and prospects for high throughput screening of drug compounds. Comb. Chem. High Throughput Screening.

[54] Świderski M., Seroka J., Guziejewski D., Krzymiński P., Miniak-Górecka A. (2025). Influence
of Electrode
Polishing Protocols, Potentiostat Models, and LOD Calculation Methods
on the Electroanalytical Performance of SWV Measurements at Glassy
Carbon Electrodes. Molecules.

[55] El-Sayed G. O., Amin A. S. (2010). Voltammetric determination
of melatonin in tablet dosage
forms and human serum. Lat. Am. J. Pharm..

[56] Sunon P., Wongkaew P., Johns J., Johns N. (2018). Characterization of
cerium oxide-chitosan nanocomposite–modified screen printed
carbon electrode and application in melatonin determination. Int. J. GEOMATE.

[57] Martínez-Moro R., Del Pozo M., Vázquez L., Martín-Gago J.
A., Petit-Domínguez (2023). Electrochemical sensor
based on the synergy between
Cucurbit [8] uril and 2D-MoS_2_ for enhanced melatonin quantification. Sci. Rep..

[58] Bekheit G. E. (2000). Determination
of melatonin by differential pulse voltammetry at modified carbon
paste electrode. Asian J. Chem..

[59] Smajdor J., Piech R., Pięk M., Paczosa-Bator B. (2017). Carbon black
as a glassy carbon electrode modifier for high sensitive melatonin
determination. J. Electroanal. Chem..

[60] Rahmati R., Hemmati A., Mohammadi R., Hatamie A., Tamjid E., Simchi A. (2020). Sensitive voltammetric
detection of melatonin in pharmaceutical
products by highly conductive porous graphene-gold composites. ACS Sustainable Chem. Eng..

[61] Santhan A., Hwa K. Y. (2022). Rational design
of nanostructured copper phosphate
nanoflakes supported niobium carbide for the selective electrochemical
detection of melatonin. ACS Appl. Nano Mater..

[62] Sebastian N., Yu W. C., Balram D., Chen Q., Shiue A. (2023). Porous hematite embedded
C and Fe codoped graphitic carbon nitride
for electrochemical detection of pineal gland hormone melatonin. Mater. Today Chem..

[63] Satianram W., Sunon P., Ngokpho B., Nijpanich S., Chanlek N. (2024). Electrochemical detection of melatonin at nano-sized
highly boron-doped diamond electrode. J. Electrochem.
Soc..

[64] Peikova L., Tzankova D., Stancheva-Zlatkova M., Zlatkov A. (2024). Development
of RP-HPLC methods for the analysis of melatonin alone and in combination
with sleep-enhancing dietary supplements. Pharmacia.

[65] Kamaris G., Mitsiou V. P. M., Chachlioutaki K., Almpani S., Markopoulou C. K. (2025). Development
and Validation of an HPLC-FLD Method for the Determination of Pyridoxine
and Melatonin in Chocolate Formulations—Digestion Simulation
Study. Chemistry.

[66] Brugnera M., Vicario-de-la-Torre M., Andrés-Guerrero V., Bravo-Osuna I., Molina-Martínez I. T., Herrero-Vanrell R. (2022). Validation
of a rapid and easy-to-apply method to simultaneously quantify co-loaded
dexamethasone and melatonin PLGA microspheres by HPLC-UV: encapsulation
efficiency and in vitro release. Pharmaceutics.

[67] Sezgin B., Soyseven M., Arli G. (2024). Greenness
assessment and comparison
of the developed and validated green HPLC-PDA, HPLC-FLD, and HPLC-ELSD
methods for the determination of melatonin in various products using
analytical eco-scale, NEMI, GAPI, and AGREE greenness metric tools. Microchem. J..

[68] Shi M., Zheng X., Zhang N., Guo Y., Liu M., Yin L. (2023). Overview of sixteen green analytical chemistry metrics for evaluation
of the greenness of analytical methods. TrAC,
Trends Anal. Chem..

[69] Vadnere N., Patel M. (2025). UV spectrophotometry and HPLC quantification
of vibegron: evaluating
whiteness, greenness, and blueness. Accredit.
Qual. Assur..

