# Supplementary material for: A Green and Sustainable Approach for Melatonin Determination: Comparative Assessment of Hybrid Micellar Liquid Chromatography and Differential Pulse Voltammetry
Source: ACS Omega. 2026 May 21;11(21):31405–17. doi: 10.1021/acsomega.6c01692 (PMC13234676; doi:10.1021/acsomega.6c01692)
Supplement: Supplementary file 1 [file ao6c01692_si_001.pdf]

SUPPORTING INFORMATION

A green and sustainable approach for melatonin determination: Comparative assessment of hybrid micellar liquid chromatography and differential pulse voltammetry

Zehra Üstün[1*], Ümran Sofu[2], İlkay Konçe[2], Ebru Çubuk Demiralay[2]

[1]Atayalvaç Vocational School of Health Services, Süleyman Demirel University, Isparta, Türkiye

[2]Department of Analytical Chemistry, Faculty of Pharmacy, Süleyman Demirel University, Isparta, Türkiye

*Corresponding author: zehraustun@sdu.edu.tr

Table S1. SST parameters and formulas

Parameters	Formulas	Explanations
**capacity factor *(k)***	$k = \frac{t_R - t_0}{t_0}$	t_R: retention time; t_0: hold up time
**selectivity factor (*α*)**	$\alpha = \frac{k_2}{k_1}$	k_1: 1. peak capacity factor; k_2: 2. peak capacity factor
**separation factor (*R_s*)**	$R_s = {}^1/_4\sqrt{N} \times (\frac{\alpha-1}{\alpha}) \times (\frac{k_2}{1+k_2})$	*N:* theoretical plate number
**tailing factor (*TF*)**	$TF = \frac{a+b}{2a}$	*TF*: tailing factor; a and b: front and back half widths at 5% of maximum peak height
**theoretical plate number (*N*)**	$N = 5.54\left(\frac{t_R}{W_{0.5}}\right)^2$	$W_{0.5}$: the peak width at half-height

Table S2. Data obtained from the φ_{EtOH}-log*k* relationship

$\varphi_{solvent}$	Linear function	Correlation coefficient (r)	*S*	k_w	φ_0
EtOH-water	log*k* = –5.546φ +1.343	0.999	5.546	22.034	0.226

φ: amount of organic solvent; *S*: solvent strength parameter; k_W: *k* value of analyte in mobile phase containing 100% water; φ_0: hydrophobicity descriptor

Table S3. Chromatographic performance data calculated using the Purnell equation under optimized conditions

Compounds	k	α	$k_2/(k_2+1)$	$\alpha - 1/\alpha$	$(1/4)\sqrt{N}$	R_s
Methylparaben (IS)/Melatonin	3.188	2.305	0.723	0.417	14.512	6.254

k: capacity factor; *α*: selectivity factor; k_2: 2. peak capacity factor; *N:* theoretical plate number; R_s: separation factor

Table S4. Comparison of the performance of selected HPLC methods for melatonin sensing in different samples.

Parameter	Proposed method	Reported method [5]	Reported method [54]			Reported method [51]	Reported method [52]	Reported method [53]
Detection method	Hybrid micellar liquid chromatography	**HPLC-fluorescence**	HPLC-PDA	HPLC-FLD	HPLC-ELSD	HPLC-UV	HPLC-FLD	HPLC-UV
Mobile phase	EtOH-water binary mixture containing 20% (v/v) EtOH adjusted to pH 7.5	A mixture of MeOH adjusted to pH 5.5 and 0.05% TEA (70:30, v/v).	Water-EtOH) (70:30, %v/v)			Mixture of ACN: water (70:30, %v/v)	water with formic acid 0.2%, and B, ACN with formic acid 0.2%	MeOH: water (70:30, %v/v)
HPLC column	Ascentis RP Amide (150 × 4.6 mm, 5 µm)	HyperClone ODS C18 column (150×4.6 mm, 5 µm)	SVEA column (250 × 4.6 mm, 5 µm)			Kinetex EVO C18 column (250 × 4.6 mm, 5 µm)	Aqua Evosphere Fortis column (250 × 4.6 mm, 5 mm)	Ascentis C18 HPLC Column (250 × 4.6 mm, 5 µm)
Retention time (min)	4.123	1.78	3.55	3.31	3.36	2.61	12.30	2.91
Column temperature	37 ^{o}C	-	40 ^{o}C			25 ^{o}C	45 ^{o}C	45 ^{o}C
Sample	pharmaceutical formulation	pharmaceutical formulation	supplementary formulations			supplements	chocolate formulation	biodegradable poly(D,L-lactic-co-glycolic) acid (PLGA) microspheres co-loaded with melatonin
**Linearity range**	2.0–8.0 µg/mL	150.0-1500 ng/mL	0.01–100 µg/mL	0.1–500 µg/mL	5–1000 µg/mL	0.0125 - 0.1 µg/mL	3.2–96 ng/mL	1.0–20 µg/mL
LOD	0.335 µg/mL	18.78 ng/mL	1.20 ng/mL	0.02 ng/mL	1.26 µg/mL	0.125 µg/mL	0.92 ng/mL	0.25 2.0–8.0 µg/mL
LOQ	1.016 µg/mL	57.17 ng/mL	4.0 ng/mL	0.07 ng/mL	4.21 µg/mL	0.250 µg/mL	3.04 ng/mL	0.76 2.0–8.0 µg/mL
Recovery%	99.570 ± 1.754	100.01±0.86*	102.06	99.78	98.58%	100%	100.5±0.80*	100.10

*: Standard deviation; EtOH: Ethanol; MeOH: methanol; ACN: acetonitrile; TEA: Triethylamine; PDA: Photo Diode Array detector; FLD: Fluorescence detector; ELSD: Evaporative Light Scattering detector; UV: Ultraviyole detector

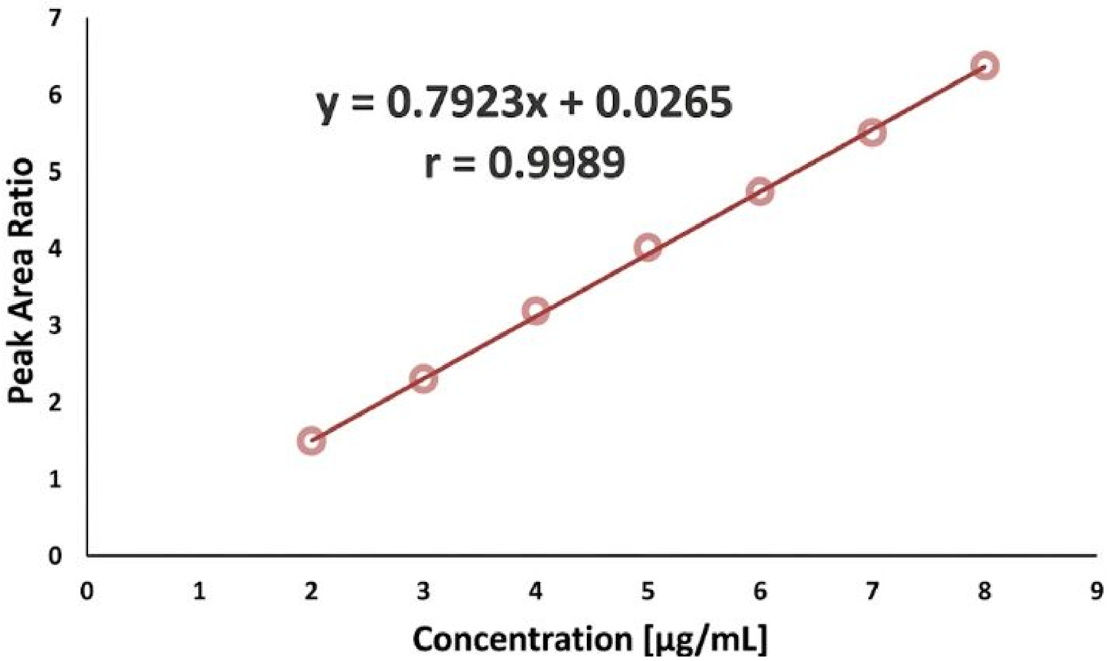

Figure S1. Calibration graph of melatonin for RPLC method

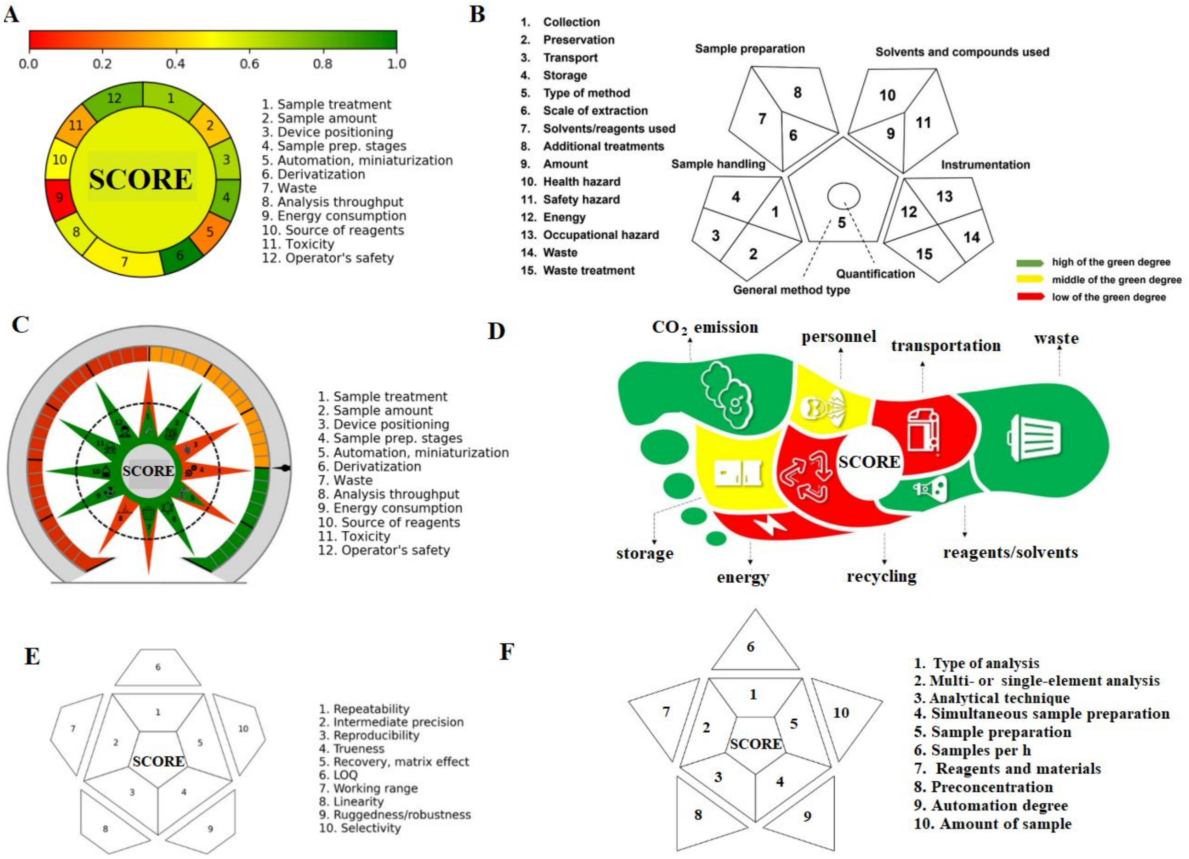

Figure S2. Tools used for evaluating greenness and whiteness in both methods A) AGREE B) GAPI C) AGSA D) CAFRI E) RAPI, and F) BAGI
